# Thriving… or Just Surviving? Autistic Journeys in Higher Education

**DOI:** 10.1007/s11920-024-01560-x

**Published:** 2024-11-07

**Authors:** Chiara Horlin, Katie Almond, Alex Bowen, Ashley Robertson

**Affiliations:** School of Psychology and Neuroscience, University of Glasgow, 62 Hillhead Street, Glasgow, G12 8QB UK

**Keywords:** Autism, Higher education, Neurodiversity, Post-secondary education, Experiences, Disclosure, Inclusion, Stigma, Workplace

## Abstract

**Purpose of Review:**

Here we synthesise key recent (2021–2024) research that aims to understand the experience of autistic people, both staff and students, who navigate the Higher Education (HE) environment.

**Recent Findings:**

Autistic students and staff continue to experience a lack of flexible, consistent and personalised support within the HE context, and tensions remain between the benefits of disclosure and the discrimination that may result. Significant missed opportunities remain for greater social, emotional and practical supports for autistic members of the HE community.

**Summary:**

Distinct barriers to both access and ‘flourishing’ for autistic people within HE remain. Personal narratives continue to describe a range of both positive and negative experiences within HE, yet it remains clear that HE may be inherently hostile in its setup, and that it takes a great deal of effort to fight against this.

**Supplementary Information:**

The online version contains supplementary material available at 10.1007/s11920-024-01560-x.

## Introduction

Until recently, autism has not been extensively explored within a Higher Education (HE) context. The reasons for this are likely manifold, but it is clear that there is an assumption that there are not many autistic people within HE. This is likely due to stereotypical tethering of autism with intellectual disability, and deeply rooted societal stigma [1, 2]. Gathering accurate data on how many autistic people there are within the wider HE community is particularly difficult due to complexities around acceptance and disclosure [3, 4], timing of diagnosis across the lifespan [5, 6], and systematic inequalities related to diagnosis [7, 8]. This not only clouds our precise understanding of ‘official’ student disability status, but also of autistic student completion rates, graduate destinations, and the experiences of autistic staff.

There have been significant increases in seeking and receiving diagnoses of autism, with UK adult autism diagnosis rates increasing from 1 in 100,000 in 1998, to 20 in 100,000 by 2018 [9], and the most recent estimated adult prevalence being around 1.1% [10]. This increase in diagnostic rates is much conjectured upon in the media [11–13], yet is likely a result of growing acceptance, and a more nuanced understanding of autism beyond narrow and rigid historical stereotypes [9]. Inevitably, increases in recognition and diagnosis will be reflected in increasing numbers of autistic students seeking and transitioning into HE. Indeed, HESA reports for the 2021/2022 academic year report over 18,820 autistic students at university in the UK compared to 6,845 within the 2014/2015 session [14]. Due to the complexities around recognition, disclosure, and a variety of barriers to diagnosis [15] it is safe to assume these statistics underestimate the true incidence [16].

The waters, unfortunately, become even murkier when trying to understand the journeys of autistic students after leaving school, with little nuanced information about transition into, progression through, or pathways beyond their undergraduate degrees into employment or further postgraduate study. Recent analysis of HESA and Graduate Outcomes Survey data (between 2012 and 2018) compared outcomes between autistic, disabled, and non-disabled graduates in the UK, finding only 34% of autistic graduates are in full-time employment compared to a rate of 57% for graduates disclosing other disabilities, and 68% disclosing no disability [16]. Autistic graduates were also more likely to earn below £20,000 than both disabled and non-disabled graduates [16]. Despite a decrease in unemployment for autistic graduates from 20% in 2012/2013 to 12% in 2017/2018 [16], Lucas and colleagues [17] found that over half of autistic graduates who were employed 1–5 years from graduation could be considered underemployed based on their roles not requiring university qualifications.

Previous reviews of the limited catalogue of literature to date describe significant challenges and barriers for autistic people traversing through HE [18–20]. Here we synthesise some key recent (2021–2024) research that aims to understand the experience of autistic people, both staff and students, who navigate the HE environment. It is clear that, although there are some glimpses of opportunity and optimism, for autistic people the landscape of HE remains precarious.

## The Experiences of Autistic Students in HE

Patterns in recent research are clear. Distinct barriers to both access and ‘flourishing’ for autistic students within HE remain and opportunities to ameliorate them are yet to be fully monopolized.

The transition to university can be jarring for anyone, let al.one autistic students who are likely to be shifting from structured and supported environments to settings rife with uncertainty and changeability. This change, alongside unpredictable social expectations, can result in an overwhelming and intimidating experience. Scott and Sedgewick [21] report that, despite the anxiety around the transition into university life, autistic students recognize and value the opportunity for personal growth, autonomy and greater independence. Nonetheless, this optimism is balanced with the reality that transition is reported as a significant trigger or exacerbator of poor mental health for autistic students [21, 22]. Although mental health challenges for autistic students may not be qualitatively different from those of neurotypical students [23], they may be more likely to be vulnerable to these issues. Indeed, 70% of neurodivergent people report co-occurring mental health diagnoses [24–27] in contrast to the lifetime and 12-month prevalence estimates of most common mental health concerns amongst HE students overall being around 30% [28]. Social connectedness, or lack thereof, can serve either as a protective or vulnerability factor at this transition point, as highlighted by the accounts within Goddard and Cook [22]. Focusing predominantly on the social experiences of autistic HE students in the UK, the authors highlight a contradiction of the stereotypical expectation that autistic students do not seek or desire peer friendships to the same degree as neurotypical students. In fact, social mentoring, particularly during key milestones such as ‘Freshers’ events (events welcoming new first year students), is seen as an opportunity to scaffold early community-building [22] and aid autistic students in finding informal community supports that are incredibly valued [21], particularly when such support is provided by neurodivergent peers [29].

Even after this critical transition period, barriers and challenges remain within the navigation of day-to-day course-related necessities. Autistic students experience inherent challenges in executive functioning and information processing that impact on the completion of coursework, with situational barriers frequently exacerbating this [29]. Despite increasing awareness of inclusive and accessible pedagogy within HE, autistic students continue to experience frustration with the lack of clarity and structure in teaching materials and the overwhelming sensory demands of university life [29]. In addition, they are often asked to learn without reasonable adjustments and supportive technologies such as the use of lecture recordings being standard practice [22]. These additional burdens often fuel existing anxiety, fatigue and fear of stigmatisation [29] which are likely further exacerbated by the despair, frustration and helplessness resulting from continual cycles of self-advocacy and repeated disclosure to academic staff who may have variable degrees of understanding or willingness to accommodate [21, 23, 30, 31].

The access to and suitability of reasonable adjustments, and the available support (both formal and informal) for autistic students remains an important focus of the limited extant literature. Overwhelmingly, recent studies reaffirm the absolute necessity for institutional support to be consistent, coordinated, and most importantly, personalized to the needs of the individual [16, 17, 21, 29, 30, 32]. Students from both the UK and US highlight that a difficulty in accessing support is often the first potential barrier that they may encounter, but even once accessed, discontinuity between services creates additional challenges [21, 30]. Services may not effectively communicate with each other, and the understanding of academic staff may not always be aligned, or may not evolve with, a student’s changing needs. Unfortunately, this feeds into the aforementioned cycle of continual self-advocacy and repeated disclosure. It is also clear that formal support alone is not usually sufficient, and is necessarily supplemented by informal peer support [21, 22, 29], emotional support from disability support providers [30], or academic staff, in a capacity beyond their formal role [32].

Another focus of recent research has been a response to the alarming disparity in successful employment transitions for autistic students graduating from university. There appears to be an urgent need for a ‘bookending’ of support, both into and out of HE as, despite often seeking support [32] autistic graduates report feeling unprepared to move into the workplace, both practically and in terms of emotional readiness in dealing with the fear and anxiety surrounding this major change [17]. Career support is often a standard service offered by HE institutions that is made available to all students, and some of the barriers to accessing this support do not appear to be unique to autistic students alone, with students of all neurotypes citing they are either unaware of services, or find services lack specificity [32]. Yet autistic students report specific problems related to the lack of structure of these services, and a lack of opportunity to rehearse the practical skills required for recruitment and employment [32, 33]. Vincent and Fabri [33] explored barriers and pathways to successful transition to employment for autistic students and identified multiple opportunities for bolstering graduate preparedness, particularly when considered as part of coordinated services within HE and beyond. Alongside practical services such as career planning and skill-based competencies to support success during recruitment and interviewing, autistic graduates noted additional barriers that were rooted in anxiety and uncertainty about the workplace culture they may encounter [33]. Although, this is likely not something career support services within HE can directly ameliorate, a wider HE culture that is neuroaffirming, inclusive, and welcoming of difference may engender confidence in autistic graduates and empower self-advocacy. This would be particularly powerful if the transition to employment was supplemented by external mentoring and individualized support.

## The Experiences of Autistic Staff in HE

Existing literature covering the experiences of autistic staff is sparse and describes a landscape for postgraduate students and staff (academic and non-academic) that is potentially bleaker than for undergraduate students. Mellifont’s [20] narrative review synthesises literature (albeit only 34 relevant studies) from 2010 to 2020 that explores the lived experience of neurodivergent HE staff sharing a balance of both positive and negative experiences. Crucially, the polarity of these experiences seems to hinge on the complexity of disclosure and the ambivalent emotions that it can generate within the autistic person. While some autistic academics experience a positive and supportive reception from both colleagues and their wider institution after disclosing, many experience systematic ableism, disempowerment, and stigmatising judgements about both professional and personal competency.

Since the period covered within Mellifont’s review, only a small number of studies or personal accounts provide any further clarity. In a study by Martin [34], all 12 participants working within UK HE had experienced barriers to obtaining and sustaining employment, citing difficulties during recruitment (which tends to rely on neurotypical communication styles), unclear expectations and unnavigable bureaucracy, a lack of willingness for peers to understand, and stigma leading to stereotyping. Paralleling some of the conclusions of previously described studies with autistic students, Martin [34] notes that autistic people are not avoidant of workplace communication but may be frustrated or disadvantaged by unclear or imprecise communication, as well as unspoken expectation. Just like autistic students in HE, autistic staff can experience a disabling level of bureaucratic complexity from the point of initial recruitment and throughout onboarding and career progression. This insight is both echoed and amplified by Jones [35] in their thematic analysis of the barriers and facilitators experienced by autistic academics. While highlighting that the competitive, demanding, and increasingly insecure nature of modern academia [36, 37] is problematic for all, Jones [35] illustrates how autistic people may be at a particular disadvantage. While a new social landscape can be overwhelming for autistic students transitioning into HE, the social and communication demands on staff are arguably more complex. The study highlights the high volume, superficial and largely transient social interactions with students that, paired with the inherent performative tension between ‘educating’ and ‘entertaining’ in HE, places huge demands on masking and managing social interactions. Similarly, communication with students, colleagues, and management, can be a precarious tightrope of balancing honesty, unspoken politics, and occasional deception in an environment that can often prioritise competition (for funding, positions, or promotion) or ‘customer’ (student) satisfaction [35].

Importantly, Jones [35] highlights that the high task demands of HE are not only potentially disabling for autistic students completing coursework, but also the autistic staff who deliver and manage it. Despite several participants emphasising the benefits of some academic roles in HE affording a degree of flexibility, self-determination, and opportunity to deeply engage in topics of interest when they are appropriately supported, being a neuro-minority within a neurotypical hegemony is inherently challenging. Participants noted that constant task-switching, high volume of information processing, perfectionism, uncertainty, and changeability take a significant toll on wellbeing, particularly when compounded by oft rigid processes and inefficient bureaucracy.

While empirical studies of the lived experience of autistic staff in HE might be thin on the ground, there are still important opportunities to hear autistic voices. Alongside personal accounts of Monique Botha [38] and Damian Milton [39], autistic staff and researchers share insight and advice within two recent papers [40, 41]. Within roundtable discussions held at INSAR [40] and the advice-gathering of autistic academics [41] there is a sense of both cautious hesitancy and strong passions, but ultimately hard-fought lessons that none wish future autistic students or staff to experience first-hand. These personal narratives not only provide useful seeds for future empirical exploration, but immediate testimony that academia may be inherently hostile in its setup, and that it takes effort to fight against this. How much effort exactly seems to depend on the individual people surrounding autistic staff, and whether the effort is worth the benefit.

## Conclusion

As the number of autistic members within the HE community will likely increase, it is important to acknowledge that the amount of research and insight into their lived experience remains limited. This is particularly true for autistic people who persist from undergraduate studies, into postgraduate programmes and possibly academic careers. It also seems no coincidence that the majority of the research included here is qualitative in nature. While this may currently be the only avenue to reflect authentic autistic voices, or even begin to capture the scope of the heterogenous need, this cannot be accepted uncritically given that much autism research (especially qualitative) may not be truly representative of diverse autistic experiences [39]. Access to funding for participatory and emancipatory research remains difficult in competitive funding structures that prioritise biological and genetic research [39, 42] but is likely essential to provide more targeted institutional, or sector-wide support. Yet despite the limited catalogue to date, there is no reason that these accounts and insights should not still feed into policy and practice to facilitate moving from ‘survival for some’ to ‘success for as many as possible’.

Although many entrenched barriers to success for autistic students and staff remain, literature highlights some opportunities for optimism, and the powerful role that autistic advocates can play. Much of the effort in pushing for change, whether at an individual or institutional level does fall upon autistic people themselves [39] which places an implicit burden on being ‘out’ and carrying the weight of other’s expectations to be an autistic ‘hero’. The importance of autistic role models in academia is obvious, yet many leave for other sectors due to hostility or burnout [38]. The largest barrier still to overcome may be systematic, institutional-level bureaucracy that prohibits fully coordinated, person-centered and individualized support. Incidences of poor awareness, misunderstanding and discrimination do still occur, as do examples of HE staff who may be willing, but not able, to accommodate autistic people due to institutional pressures or attitude-behaviour gaps [31].

The most recent research highlights implementable and practical opportunities that unfortunately feel like singing from the same old songbook. As a priority, autistic students need clear, accessible and well-structured learning materials alongside individualised support, with continuity both between services, and throughout the various stages of their HE experience. These measures should be core responsibilities for HE providers, yet institutional pressures around ever-increasing student numbers, and demands on staff capacity relegate such practices to the realm of ‘reasonable adjustments’, rather than ‘basic rights’. In addition to this, there is a need for holistic recognition, not only of the individual and their combined academic and socio-emotional needs, but their journey into and beyond HE. Similarly, staff experiences should include a streamlined continuity from initial recruitment to a welcoming immersion into an appropriately flexible and neuro-affirming workplace culture that understands need for clarity, transparency, and patience, as well as the depth of potential that autistic staff can contribute when well-supported. Whilst in the immediate term, navigating HE may continue to be a matter of ‘survival’ for many, we must continue to nourish any and all optimism that supports autistic students and staff to see value in their unique contribution, and confidence in their being capable of making a positive impact both within and beyond the HE context.

## Electronic Supplementary Material

Below is the link to the electronic supplementary material.

Supplementary Material 1

Supplementary Material 2

Supplementary Material 3

Supplementary Material 4

## Data Availability

No datasets were generated or analysed during the current study.
